# Primary Pericardial Angiosarcoma: A Case Report and Review of Literature

**DOI:** 10.1186/1749-8090-10-S1-A36

**Published:** 2015-12-16

**Authors:** Chakrabarti Amitabha, Biswarup Purkayastha, Lokesh Kukatla, Sanjib K Pattari

**Affiliations:** 1 Department of Cardio Thoracic and Vascular Surgery, NH Rabindranath Tagore International Institute of Cardiac Sciences, Kolkata, India; 2 Department of Cardio Thoracic and Vascular Surgery, KPC Medical College, Kolkata, India; 3 Department of Radio Diagnosis, NH Rabindranath Tagore International Institute of Cardiac Sciences, Kolkata, India; 4 Department of Pathology, NH Rabindranath Tagore International Institute of Cardiac Sciences, Kolkata, India

## Background/Introduction

58-year-old diabetic, ex-smoker with shortness of breath, recurrent pericardial effusion with impending cardiac tamponade was diagnosed with a pericardial mass on computed tomography, underwent surgical resection (partial pericardiectomy). Histopathology and immuno-histochemistry clinched the diagnosis of Pericardial Angiosarcoma. He refused further treatment.

## Aims/Objectives

To augment our understanding of Cardiac and Pericardial Angiosarcoma and formulate a proper investigation workup plan including imaging modality of choice and multi-modality management plan.

## Method

PubMed and Google were searched for primary references using the terms "Cardiac Angiosarcoma" "Pericardial Angiosarcoma" "Treatment of Angiosarcoma" and "Newer Therapies in Angiosarcoma". The Case Reports, Case Series and Review articles obtained were thoroughly reviewed.

## Results

Shortness of Breath remains the most common symptom with Trans Thoracic Echocardiography as Investigation of Choice, but Computed Tomography(CT) and Magnetic Resonance (MRI) being of more yield.

The disease shows a distinct male preponderance with 64.2% being male. Mean age of presentation being 44.15 ± 19.65 years. Surgical Resection - alone or in conjunction, was done in 85.7% (reviewed in Table 1), 1 patient was managed conservatively, 1 patient refused treatment. Novel treatments have shown promise demanding further research

## Discussion/Conclusion

Cardiac and Pericardial angiosarcoma are rare, aggressive neoplasm often presenting late with loco regional metastasis, thus jeopardising operability and prognosis. Echocardiography is used most commonly, in addition HRCT(High Resolution CT) and MRI have been used for better cross sectional imaging. And 18 FDG PET-CT(Positron Emission Tomography - Computed Tomography) apart from being an useful adjunct, possibly would become the imaging modality of choice in coming decades. Surgical Resection - alone or in conjunction, remains the most commonly used treatment modality. Novel treatments with monoclonal antibodies and chemotherapeutic agents have shown excellent results and merit further research.

## Consent

Written informed consent was obtained from the patient for publication of this abstract and any accompanying images. A copy of the written consent is available for review by the Editor of this journal

